# DISBAC₂(3) fluorescent probes illuminate new paths for screening stress-resistant crops

**DOI:** 10.1093/plphys/kiaf522

**Published:** 2025-10-13

**Authors:** Tengteng Shi, Jiawen Zhang, Ning Zhang

**Affiliations:** State Key Laboratory of Wheat Improvement, College of Agronomy, Shandong Agricultural University, Tai’ an, Shandong 271018, China; State Key Laboratory of Wheat Improvement, College of Agronomy, Shandong Agricultural University, Tai’ an, Shandong 271018, China; State Key Laboratory of Wheat Improvement, College of Agronomy, Shandong Agricultural University, Tai’ an, Shandong 271018, China; Assistant Features Editor, Plant Physiology, American Society of Plant Biologists

With the intensification of extreme climates, crops are increasingly exposed to various adverse environmental conditions, leading to reduced yields and deterioration in quality (Liu et al. 2023; Qi et al. 2024). Therefore, plant breeders should be selecting and cultivating stress-resistant crops (Benitez-Alfonso et al. 2023).

Membrane potential (MP) refers to the voltage difference between the interior and exterior of the cell, typically negative under normal conditions due to ion distribution across the plasma membrane, and serves as a central electric gradient that drives the uptake and translocation of nutrients. However, upon exposure to environmental stress, ion channels on the cell membrane become inactivated, leading to a depolarized state of the MP (Hedrich and Geiger 2017; Zeng et al. 2024). Stress-resistant crops can better maintain normal cellular metabolic activities under adverse conditions while exhibiting minimal changes in MP (Shabala et al. 2016). Therefore, the magnitude of change in their MP can be used to determine the stress resistance of crops.

The traditional method for detecting MP is to insert a microelectrode into a single cell, a process that is time consuming and labor intensive, has low throughput, and requires skill (Liu and Miller 2020). Another method utilizes voltage-sensitive fluorescent probes, such as DiSBAC₂(3) (bis-(1,3-diethylthiobarbituric acid) trimethine oxonol), to assess MP changes by monitoring fluorescence intensity. DiSBAC₂(3) is a negatively charged organic molecule. Under resting MP, it remains outside the cell, resulting in low fluorescence. When the membrane depolarizes, DiSBAC₂(3) enters the cell and associates with phospholipids on the plasma membrane, resulting in high fluorescence (Serre et al. 2021).

Recently in *Plant Physiology*, Ivsic et al. (2025) investigated the potential to use DiSBAC₂(3) as a sensitive, high-throughput, and reliable alternative for screening stress-resistant crops. First, they showed that results from the DiSBAC₂(3) dye method matched those obtained with the electrode impalement method for detecting NaCl-induced MP changes in *Tradescantia virginiana* root.

The authors next tested the probe efficacy under 3 different types of treatments. First, DiSBAC₂(3) fluorescent probe demonstrated high reliability in detecting salt stress–induced membrane depolarization across various plant species (*Arabidopsis*, barley, rice, pea, and corn) when validated against conventional microelectrode impalement. A strong correlation was observed between fluorescence intensity changes and actual MP shifts—for instance, a 35-mV depolarization in rice corresponded to a 1.7-fold increase in fluorescence intensity. Notably, as root size and thickness influence fluorescence signals, species-specific calibration of fluorescence-to-MP relationship is essential for accurate measurements, unless confocal scanning microscopy is used.

Second, the researchers employed the DiSBAC₂(3) fluorescent probe to detect membrane depolarization in barley roots under hypoxia stress, a condition that commonly arises as a physiological consequence of waterlogging. This technique effectively distinguished between hypoxia-sensitive (Naso Nijo) and -tolerant (TX9425) varieties (Figure 1). The fluorescence intensity increased significantly in the sensitive variety after stress, while no significant change was observed in the tolerant variety. These results were highly consistent with electrophysiological measurements, providing a reliable tool for the rapid screening of waterlogging stress-tolerant germplasm resources.

**Figure 1.**
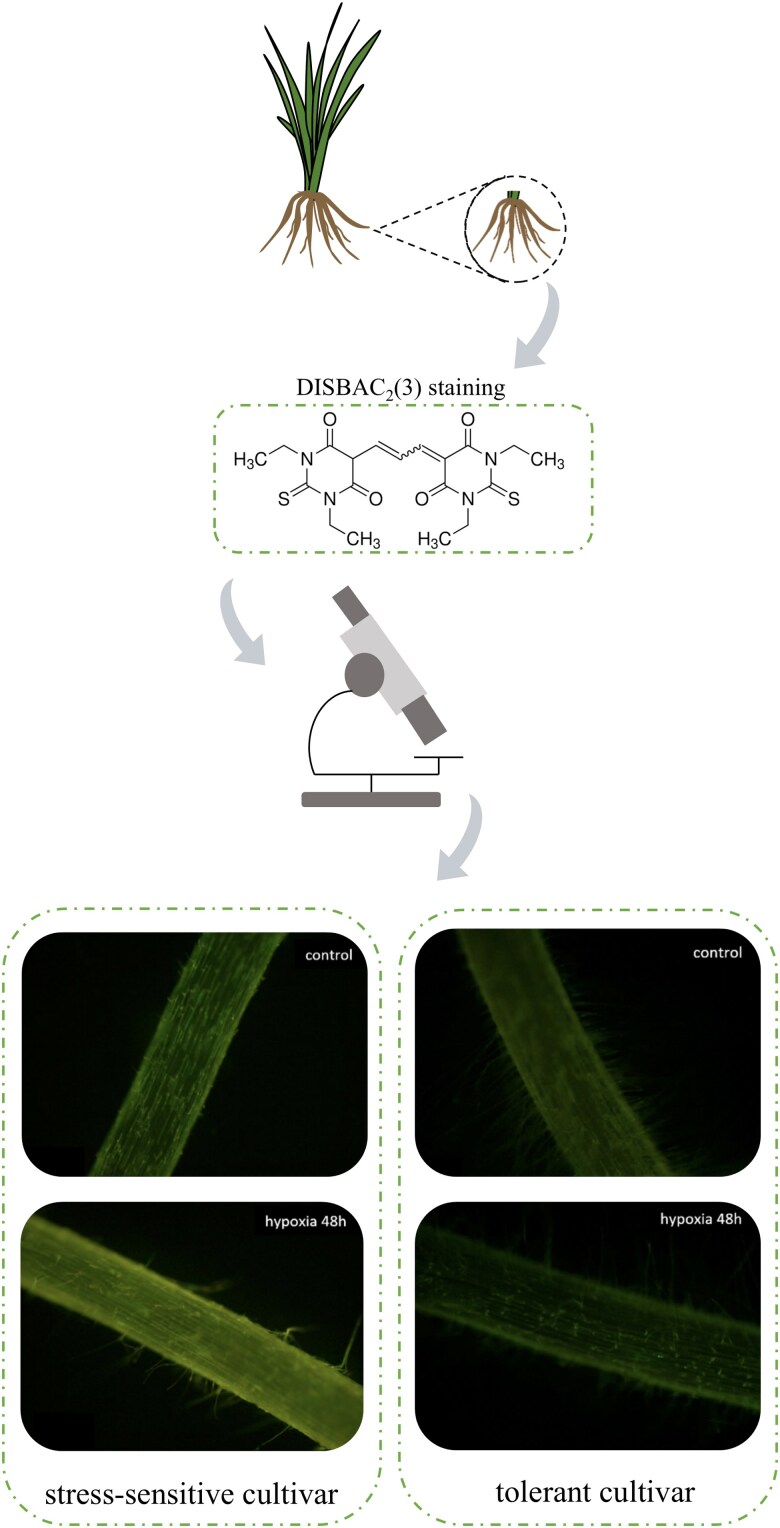
Voltage-dependent fluorescence response of DiSBAC₂(3) under stress versus control conditions (summarized from Ivsic et al. 2025). Shown are a schematic of the chemical structure of DiSBAC₂(3) and representative fluorescence images of barley roots under control and hypoxia conditions. Control roots show low fluorescence, whereas hypoxia-treated roots exhibit increased fluorescence in sensitive genotypes but minimal changes in tolerant genotypes, highlighting the ability of DiSBAC₂(3) to distinguish stress responses.

Last, the authors utilized the DiSBAC₂(3) fluorescent probe to detect light-induced hyperpolarization in guard cells. By applying a 20-mM NaCl pretreatment to induce slight membrane depolarization, the probe effectively captured the time-dependent hyperpolarization trend correlated with stomatal opening. The results were highly consistent with proton flux assay data, confirming the H⁺-ATPase activation mechanism. Taken together, these findings demonstrate the broad applicability of DiSBAC₂(3) for detecting both membrane depolarization and hyperpolarization across multiple species under various stress conditions.

These studies highlight the potential uses for DiSBAC₂(3) in plant breeding. First, in phenotyping platforms, this probe could be incorporated into automated imaging pipelines for high-throughput screening of diverse germplasm collections, enabling breeders to rapidly identify promising lines under controlled stress assays. Second, in functional genomics, DISBAC₂(3)-based measurements could be combined with CRISPR/Cas mutagenesis or transcriptomics to dissect the role of stress-related genes in maintaining MP homeostasis. Third, in field applications, the development of portable imaging devices or sensor-based platforms could allow real-time monitoring of MP dynamics in crops growing under natural conditions. Such data could serve as an early diagnostic signal of stress onset, informing precision irrigation, fertilization, or stress-mitigation strategies before visible symptoms appear. Finally, by coupling DiSBAC₂(3)-derived physiological measurements with machine learning and predictive modeling, it may be possible to create integrative frameworks that link genotype, physiology, and environment, ultimately guiding the design of resilient cropping systems under climate change. Together, these future applications highlight the versatility of the DiSBAC₂(3) fluorescent probe—not only as a research tool but also as a practical bridge between molecular physiology and sustainable crop production.

## Data Availability

Data availability statement is not applicable.
